# Cardiac MRI predictors for development of atrial fibrillation or flutter in patients with hypertrophic cardiomyopathy

**DOI:** 10.1186/1532-429X-17-S1-P281

**Published:** 2015-02-03

**Authors:** Helen Hoi Lam She, Michael Pak Hei Chan, Ka Fai Johnny Ma

**Affiliations:** Radiology, Princess Margaret Hospital, Hong Kong, Hong Kong; Medicine, The University of Hong Kong, Hong Kong, Hong Kong

## Background

In patients with hypertrophic cardiomyopathy (HCM), atrial fibrillation (AF) or flutter remains the most common disease complication and arrhythmia in patients with hypertrophic cardiomyopathy (HCM), occurring in up to 25% of the patients, which is 4-fold more common than the general population. Occurrence of AF in HCM, not only will lead to its notorious complications of ischemic stroke and systemic embolization, can cause symptomatic deterioration due to a significant reduction in preload. In addition to transthoracic echocardiography, cardiac MR (CMR) has played an important role in making the diagnosis as well as risk-stratifying the patients. The purpose of this study is to determine CMR predictors in patients with HCM for predicting occurrence of incident AF.

## Methods

All 58 consecutive patients with HCM, in sinus rhythm with no prior evidence of atrial arrhythmia who had CMR performed from 2008 to 2012, were identified. Patients' background demographics and various CMR parameters including maximum left atrial (LA) volume (LAmax), minimum LA volume (LAmin), LA ejection fraction, left ventricular (LV) ejection fraction, LV end diastolic and systolic volumes, LV mass, maximal septal thickness, and percentage of LV scar were prospectively recorded in our database. Patients were followed up for development of new onset atrial fibrillation as confirmed by ECG and/or Holter. Univariate analyses followed by multivariate logistic regression were used to assess the clinical impact of individual clinical and CMR parameters for predicting the development of AF.

## Results

58 patients (mean age 53.4 +/- 15.2; male sex: 58%) with diagnosis of 1) asymmetrical HCM 77%, 2) apical HCM 22.6%, 3) other forms of HCM 6.5%, were recruited. During a mean follow up of 4.1+/-0.8 years, 18 (31.0%) developed AF. When adjusted for covariates in our model, age, hypertension, minimum and maximum LA volumes (LA min, LA max) and LA ejection fraction (LAEF) were independent predictors for development of AF. Receiver operator curve were generated for minimum and maximum LA volume, and LA ejection fraction, with no significant differences between the areas under curve (0.81, 0.79 and 0.76 respectively, p=0.11). Cut off points for greatest likelihood for AF occurrence would be LA min of 16ml/m^2^, LA max of 28ml/m^2^ and LAEF <33%.

## Conclusions

LA ejection fraction, LA minmum and maximum volume on MRI are independent predictors of new onset of AF in patients with underlying hypertrophic cardiomyopathy.

